# Uniportal VATS Coil-Assisted Resections for GGOs

**DOI:** 10.1155/2019/5383086

**Published:** 2019-05-12

**Authors:** Maria Teresa Congedo, Roberto Iezzi, Dania Nachira, Anna Rita Larici, Marco Chiappetta, Lucio Calandriello, Maria Letizia Vita, Elisa Meacci, Venanzio Porziella, Mahmoud Ismail, Riccardo Manfredi, Stefano Margaritora

**Affiliations:** ^1^UOC di Chirurgia Toracica, Fondazione Policlinico Universitario A. Gemelli, IRCCS, Rome, Italy; ^2^Università Cattolica del Sacro Cuore, Rome, Italy; ^3^UOC di Radiologia Diagnostica e Interventistica Generale, Fondazione Policlinico Universitario A. Gemelli, IRCCS, Rome, Italy; ^4^Klinikum Ernst von Bergmann, Academic Hospital of the Charité – Universitätsmedizin, Humboldt University Berlin, Potsdam, Germany

## Abstract

**Backgrounds:**

Although uniportal video-assisted thoracic surgery (VATS) theoretically allows the direct palpation of any zone of the lung through a small incision, sometimes it can be difficult to localize pure ground-glass opacities anyway. The aim of this study is to evaluate the usefulness and safety of preoperative computed tomography (CT)-guided microcoil localization of GGO nodules in patients undergoing uniportal VATS lung resection.

**Methods:**

The clinical data and CT images of 30 consecutive patients (30 pulmonary nodules) who underwent preoperative CT-guided coil localization and subsequent uniportal VATS resection, from January 2017 to October 2018, were reviewed.

**Results:**

All the CT-localization procedures have been performed with success (30/30) and the mean procedure time was 35±15 minutes. The mean size of the nodules was 15,53±6,72 mm, and the mean distance of the nodules from the pleural surface was 19,08±12,08 mm. Eleven nodules (36,7%) were pure ground-glass opacities and 19 (63,3%) were mixed ground-glass with a solid component of 50% or more. In 5 cases, the localization procedure was complicated by asymptomatic pneumothoraxes and in 1 case the pneumothorax required chest tube insertion. In any case a conversion to thoracotomy was avoided because all nodules were identified and resected through uniportal VATS.

**Conclusions:**

Preoperative CT-guided coil localization seems to be a feasible, safe, and accurate procedure. It makes uniportal VATS an easy approach even for resecting small, deep, and impalpable nodules.

## 1. Introduction

With the increasing use of chest high-resolution computed tomography (CT) and the implementation of lung cancer screening programs, it has been becoming more and more common to detect ground-glass opacities (GGOs) suspected to be slow-growing lung cancers.

The subsolid aspect of these lesions makes bronchoscopy or percutaneous CT-guided biopsy unsuitable to provide a cytological examination useful to define the presence of tumor cells preoperatively or, even more difficult, the invasiveness of the tumor as defined by adenocarcinoma classification [1]. Therefore, the low accuracy and rate of false negative biopsies make the surgical excision necessary.

Extension of surgery is still under discussion because some authors believe that limited surgery (wedge resection or segmentectomy rather than pulmonary lobectomy) is more indicated for the indolent-growth lesions [2, 3].

It seems that the oncologic outcome of segmentectomy is similar to lobectomy in cT1a N0 M0 NSCLC patients [4–7], but there has not been agreement among the authors till now.

In all high volume thoracic oncologic centers, major resections for early stage lung cancer are performed using minimally invasive techniques (triportal, biportal, or uniportal VATS or robotic-assisted thoracic surgery (RATS)).

The uniportal technique seems to provide a lot of advantages, in terms of major comfort and lower postoperative pain and morbidity [8] and a good palpation of lung parenchyma compared to triportal techniques. However, sometimes it can be very difficult to palpate and identify subsolid and deep nodules even by uniportal VATS. Although there are many techniques for preoperative localization of pulmonary nodules [9, 10], CT-guided hookwire localization can be a valid alternative and an appropriate method in case of patients with solitary subsolid pulmonary nodules [11–14].

The aim of this study is to describe and review our experience with uniportal VATS resection of GGOs localized using preoperative CT-guided technique by microcoils placed into the lesion with the distal tail above the visceral pleura surface and the proximal end immediately beneath the nodule without the use of fluoroscopy.

## 2. Materials and Methods

The clinical and surgical data of 30 patients undergoing uniportal VATS resection after CT-guided microcoil localization of GGOs, between January 2017 and October 2018, were retrospectively reviewed.

The inclusion criteria based on CT scan characteristics of the lesions were (a) pure GGO or lesion with minimal solid component; (b) maximal long axis diameter < 20 mm; and (c) distance from visceral pleura > 5 mm. The surgical indications for these patients included enlargement of the nodule size or persistence of a nodule with a solid component ≥ 5 mm at CT follow-up, or positive clinical history of multifocal tumors.

All the cases were discussed by two thoracic surgeons (S.M. and M.T.C), if they considered that the nodules were not likely to be visualized intraoperatively during VATS, and one interventional chest radiologist (R.I) to determine the feasibility of microcoil localization before uniportal resection.

The main contraindications identified for microcoil localization were previous chemical pleurodesis, nodules close to the hilar structures, and severe bullous disease increasing the risk of pneumothorax.

### 2.1. Radiological Technique

The CT-guided coil localization was performed under local anesthesia.

**Figure 1:**
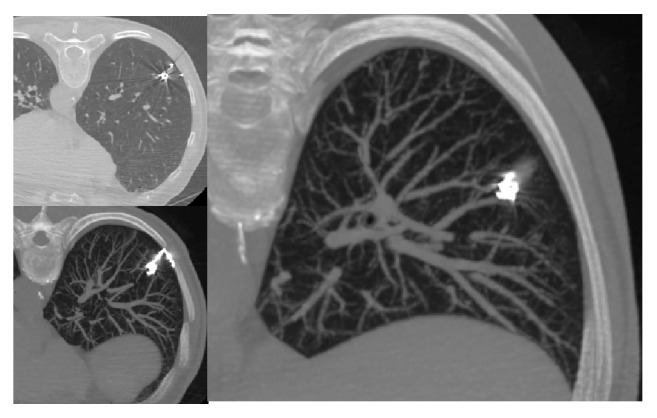
CT-images showing nodule localization by microcoil in different scanning planes.

**Figure 2:**
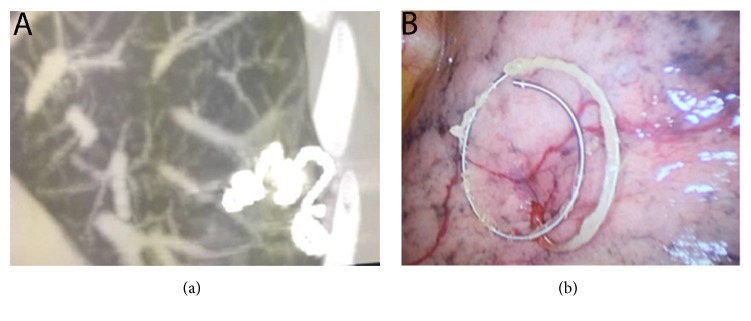
Radiological (a) and intraoperative (b) view of the microcoil deployed partially inside the nodule and partially along the visceral pleura.

On the day of operation, an interventional radiologist (R.I. with 15-year experience) performed the CT-guided percutaneous embolization-coil localization of the GGO. In detail, after the patient was placed on the CT table in a suitable position (supine or prone according to the localization of the lesion), CT scan was obtained to identify the nodule, plan the access route, also based on surgical approach, and determine the transpulmonary needle route and the distance of the nodule from the nearest pleural surface (Figure 1). After sterilization of skin around the puncture site and local anesthesia (1% lidocaine), a percutaneous 18 G needle (18 G-150 mm) was introduced from the point marked on the skin determined from the calculated length into the nodule. Once the needle tip was identified as within the nodule, the stylet was removed from the needle and an embolization coil (14-mm diameter x 14 cm length, synthetic fiber-coated, stainless steel, Cook, Bloomington, IN, USA) was pushed into the needle by the stylet. The coil was deployed beneath the nodule and partially along the transpulmonary route till the pleural space, with the distal tail of the coil left above the visceral pleura surface, serving as a guidance for the surgeon (Figure 2). After removal of the needle, subsequent CT scans were obtained for the identification of the coil deployment and its final position and eventual complications, such as pneumothorax or intraparenchymal hemorrhage.

### 2.2. Surgical Technique

The patient was placed in lateral decubitus position with arms flexed and stretched toward the head on the surgical table, with surgeon and his assistant standing in front of the patient, looking at the same screen. The procedure was performed under general anesthesia and double lumen intubation. The 2–3 cm single incision has been usually made in the 5th intercostal space but, sometimes, upper lesions required an incision in the 4th intercostal space for their better exposure and management. The incision was made according to muscle-sparing technique principles: a wound protector was useful for having more space for instruments, for avoiding soiling of the camera and for preventing the risk of wound contamination and infection. The 10 mm 30° thoracoscope was always introduced in the upper part of the incision [15].

Once the thoracoscope was in the chest cavity, the distant end of the microcoil, rolled on the visceral pleural surface, was immediately visualized by the surgeon. An endostapler was used to perform a wedge resection including the nodule marked by the coil; if necessary, palpation to confirm the presence of the microcoil into the specimen with wide free borders can be performed easily.

**Figure 3:**
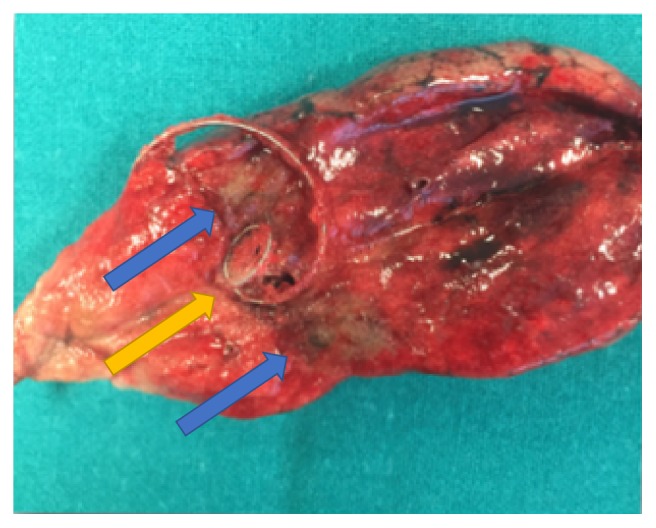
Appearance of the microcoil (yellow arrow) inside the specimen sectioned by the pathologist (blue arrows indicate the nodule sectioned).

In general, lesion was firstly removed by thoracoscopic wedge resection, and resected specimen was sent for frozen section examination immediately (Figure 3). The location of the microcoil close to the lesion has the advantage that the nodule is not disrupted for pathologist's examination. If the pathology suggested a benign or a subcentimetric carcinoma in situ with safe margins, the wedge resection was the final resection and the chest was closed with a chest tube placed through the same incision. Additional systemic lymph node sampling was performed for minimally invasive adenocarcinomas. In cases of invasive carcinomas, the operation continued with a completion lobectomy and a systematic lymph node dissection, if the patient was fit for undergoing a lobectomy. In case of previous contralateral lobectomy or multifocal tumors, only a wedge resection was performed.

### 2.3. Pathology

For the histological diagnosis, pathologists referred to the International Association for the Study of Lung Cancer/American Thoracic Society/European Respiratory Society International Multidisciplinary Classification of Lung Adenocarcinoma [1]. Lung carcinomas were classified as preinvasive lesions (including atypical adenomatous hyperplasia (AAH) and adenocarcinoma in situ (AIS)), minimally invasive adenocarcinoma (MIA), and invasive adenocarcinoma (IA).

### 2.4. Statistical Analysis

Categorical variables are reported as n (%). Continuous variables are expressed as mean ± standard deviation (SD).

Statistical analysis was performed using PASW Statistics for Windows, Version 18.0 (SPSS Inc., Chicago, IL, USA).

## 3. Results

All 30 patients were asymptomatic and the nodules were discovered occasionally at chest CT performed for other reasons or during screening program or oncological surveillance. Eighteen (60%) patients were males and 12 (40%) females, with a mean age of 63.30±17.35 years. Seven (23,3%) patients were former smoker, and 15 (50%) were current smokers.

### 3.1. Radiological Characteristics

Among the 30 resected lesions, there were 11 (36,7%) pure GGOs and 19 (63,3%) part-solid nodules. The mean diameter of all 30 lesions was 15.53±6.72 mm. The lesions were in the right upper lobe (n=10; 33,3%), right lower lobe (n=5; 16,7%), left upper lobe (n=9; 30%), left lower lobe (n=4; 13,3%), and middle lobe (n=2; 6,7%).  Table 1 shows general information of all resected GGOs.

All 30 (100%) subjects were successfully marked by coils (Figures 1 and 2), placed very close to the lesions. The mean duration of the procedure was 32 ± 15 minutes.

The mean distance between the lesion and parietal pleural surface was 20.00±12.68 mm. The mean length of needle tract was 72.58±11.99 mm. Seven minor complications (23,3%) occurred after coil placement, including 5 slight pneumothoraxes (16,7%), 1 intraparenchymal hemorrhage (3.3%) that required no intervention, and a vagal reaction (3.3%) treated by atropine administration. One patient experienced a moderate symptomatic pneumothorax requiring pleural drainage (3,3%). No other severe complications were observed, neither dislodgement of the coil.

### 3.2. Surgery

All 30 coil-labeled lesions were identified and resected by uniportal VATS with no conversion to thoracotomy. Twenty-six patients underwent a wedge resection. In subsolid nodules (19 cases) an intraoperative frozen section was required for 11 cases (36.7%): 7 of them had an invasive adenocarcinoma at frozen diagnosis, and for this reason a completion lobectomy was performed; in the other 4 cases it was not indicated because of inflammatory process. Among 26 (86.7%) patients who underwent a wedge resection, 8 (26.7%) were GGOs and 7 (23.3%) patients had multifocal forms or previous contralateral lobectomy.

Four (13.3%) patients (3 GGOs and 1 subsolid lesion) underwent an anatomical segmentectomy, directly.

No severe complication occurred during or after VATS.

The mean hospital length of stay after VATS was 5.00±4.10 days.

### 3.3. Pathology

The pathologic definitive examination revealed the following: 2 (6.7%) AIS, 4 (13.3%) MIA, 3 (10%) AAH, 2 (6.7%) invasive adenocarcinoma G1, 15 invasive adenocarcinoma G2 (50%), 3 (10%) interstitial fibrous tissue proliferation, and 1 (3.3%) fibrosis after stereotactic radiotherapy (Table 2).

## 4. Discussion

Thanks to the wide use of multidetector CT scans in screening or surveillance of lung cancer, GGOs have been being encountered increasingly.

Pure GGOs are usually difficult to be detected by VATS without previous localization, neither the pathologists can find target lesions in the frozen sections easily.

The GGOs, in particular nonsolid or part-solid nodules, are invisible at pleural inspection and are not easily palpable even in uniportal surgery, in particular in incomplete collapsed lung in patients with emphysema or in case of nodules located deeply under pleural surface.

GGO is defined as a hazy opacity that does not obscure the underlying bronchial structures or pulmonary vessels on high-resolution CT scans. The presence of a GGO component might have a notable impact on a favorable prognosis even in clinical stage IA radiologic invasive NSCLCs [16]. Therefore, a clear distinction between pure ground-glass and part-solid nodules findings on thin-section computed tomography is extremely important when evaluating the oncologic outcomes of radiologically solid NSCLCs [16].

Although finger palpation is the simplest method for localizing lesions during surgery, and uniportal VATS is the most fit for this purpose among other VATS techniques, it does not allow one to feel by finger palpation subcentimetric nonsolid tumors. According to a series by Suzuki [17], when the nodule is < 10 mm in diameter and further than 5 mm from the pleural surface, the rate of conversion to thoracotomy is about 63%. Therefore, an accurate preoperative localization of small and faint lesions is important to avoid thoracotomy conversion in VATS surgery [13].

In our Department of Thoracic Surgery in Fondazione Policlinico “A. Gemelli” IRCCS – Università Cattolica del Sacro Cuore of Rome, since May 2016, uniportal VATS replaced the open standard technique and triportal VATS in several procedures [15], up to major anatomic lung resections. This technique allows performing GGO resection through 2-3 cm incision and, in case of invasiveness of the lesion at fresh frozen, making a uniportal VATS completion lobectomy.

In the last decades, various techniques have been developed for facilitating VATS resections of pulmonary nodules. Intraoperative imaging techniques include intraoperative ultrasonography [18], CT fluoroscopy [19], electromagnetic navigation bronchoscopy [20–22], and combined technique in hybrid theatre [23, 24], but the limit of these procedures is the necessity of special equipment and professional training. Other authors proposed a lot of techniques including preoperative injection of drugs, dyes [21, 25–27], radionuclides [19, 28, 29], and contrast medium injection (lipiodol, barium) [30–32]. The disadvantages of these procedures are the necessity to perform surgery immediately after localization; in patients with silicosis, color might be difficult to visualize.

Barium could influence the diagnosis because of the inflammatory changes caused in lung parenchyma. Lipiodol has the advantage that it can be retained up to 3 months after injection and it diffuses to a very small area in lung, but it required the use of fluoroscopy [32].

In a recent review, Park [9] compared the success and complication rates associated with hookwire, microcoil, and lipiodol localization for video-assisted thoracoscopic surgery (VATS): even if all the methods had successful targeting rates, lipiodol localization seemed to have the highest overall success rate and microcoil localization the lowest complication rates.

Along with the application of various localization methods and the accumulation of operating experience, numerous reports have recognized gradually that physical methods such as wire localization showed excellent superiority [11–14, 33, 34]. Thus, the insertion of a hookwire under CT guidance seems to remain the most convenient and precise technique to localize small nodules.

However, in VATS, dislodgement occurred more frequently during lung manipulation compared to thoracotomy because of the limitations of 1-3 small incisions and use of thoracoscopic instruments for lung manipulation. Therefore, traditional CT-guided hookwire localization is not so ideal for VATS technique, like uniportal VATS.

Microcoils used for vessels embolization have some advantages: they are commonly used, easy to acquire, and inexpensive compared with radionuclides; they can also be sustained safely in the human body for days; after implantation, coils can be felt like a certain degree of hardness in the lung parenchyma and they are radiopaque, all aspects that enable finding the position by visual inspection, palpation, and, if required, fluoroscopy during surgery; the placement operation is not complicated and has good repeatability [34].

The technique we use is similar to the microcoil localization described by Sui et al. [34]; unlike Sui, we did not use either finger palpation or fluoroscopy because in all the cases the distal part of the coil was left outside the pleural surface for allowing its identification by only visual inspection (Figure 2).

Therefore, there was no requirement for involvement of facilities such as a fluoroscope, radiotracer, radioprobe, hookwire, or contract injection. Furthermore, surgeons did not receive any radiation exposure.

To the best of our knowledge, this is the first report describing the use of microcoil placed with the distal tail above the visceral pleura surface and with the proximal end immediately beneath or very close to the nodule; furthermore, unlike the other reports [14, 27], we resected all the nodules without the use of fluoroscopy. With this technique, the microcoil is immediately visualized by only inspection during uniportal VATS without any conversion to thoracotomy and any case of microcoil dislodgement. This is our preliminary experience with a small cohort of 30 consecutive patients affected by GGO nodules, and the study presents several limitations. First of all, it includes a small number of patients and it is a retrospective study not comparing other preoperative localization techniques. We think that large series of patients and multicenter randomized trial are needed to compare the different methods of localization for GGOs with the aim of identifying the most effective technique.

## 5. Conclusions

Preoperative CT-guided microcoil localization is a safe and not expensive procedure. It seems to be feasible without the use of fluoroscopy and permits the detection of GGO opacities in uniportal VATS without the need to convert to thoracotomy.

## Figures and Tables

**Table 1:** Characteristics of GGO nodules.

Characteristics of nodules	
Pure/ subsolid	11 (36.7%)/19 (63.3%)

Total diameter (mm)	15.53±6.72

Distance (lesion - parietal pleura) (mm)	20.00 ± 12.68

Lobe	
Right	
Upper	10 (33.3%)
Medium	2 (6.6%)
Lower	5 (16.7%)
Left	
Upper	9 (30%)
Lower	4 (13.4%)

Type of resection	
Wedge resection	19 (63.3%)
Segmentectomy	4 (13.3%)
Wedge resection + Completion lobectomy	7 (23.4%)

**Table 2:** Final pathological characteristics of nodules.

Pathology	30 (%)
Preinvasive lesions	
Atypical Adenomatous Hyperplasia *(AAH)*	3 (10%)
Adenocarcinoma in situ (*AIS)*	2 (6.7%)

Minimally invasive lesions	
Minimally invasive adenocarcinoma (MIA)	4 (13.3%)
Invasive adenocarcinoma	17 (56.7%)

Other	
Fibrosis	4 (13.3%)

## Data Availability

The data used to support the findings of this study are available from the corresponding author upon request.
